# Foreign Correspondence

**Published:** 1854-09

**Authors:** Austin Flint

**Affiliations:** Professor of the Theory and Practice of Medicine in the University of Louisville


### MEDICINE AND SURGERY.

SEPTEMBER, 1854. NEW SERIES. VOL. II.—NO. 9.

### ART. I.—FOREIGN CORRESPONDENCE.

BY AUSTIN FLINT, M. D., PROFESSOR OF THE THEORY AND PRACTICE OF MEDICINE IN THE UNIVERSITY OF LOUISVILLE.

*Note to the Reader:*—[This letter, the first written by Dr. FLINT after his arrival in Paris, reached us after the letter contained in the August number was in type. But although so much delayed, we doubt not that it will be as interesting as if it were fresh from the pen of its writer.—ED.]

PARIS, April, 1854.

DEAR DOCTOR:—

I have not forgotten, even amidst the many attractive novelties which surround the stranger newly arrived in the gay capitol of France, my promise to send you letters from time to time, during my sojourn in Paris, and I hasten to give you the first proof of my mindfulness of this obligation. I have now been here but a fortnight. I have seen much in this short space of time, so much that I can hardly realize the period to have been so brief; but I have seen only a small part of all that the city contains to interest the medical observer. My time therefore has been chiefly occupied with visits at the different hospitals, and hearing different lectures. To-day I commence a regular course of observations at the hospital *St. Louis*, under a private arrangement with one of the *internes.* Many of the hospitals I have not yet been able to visit, and many of the distinguished medical *savans* are yet to be seen and heard. To-day I will write of various matters *en melange*, and hereafter, if I am able to carry out my present intentions, I shall endeavor to follow some order in the subjects to which my letters will relate.

On my arrival at Paris I found that the veteran surgeon ROUX, had just died. The funeral obsequies had taken place but a few days before. The following notice of this event I translate from the *Press Mêdicale*, April 1st:—"France has lost one of those who have contributed most to her surgical glory. M. ROUX, who but lately seemed full of health and vigor, although he had arrived at an age rarely attained; M. ROUX has been, as it were, unexpectedly removed from the elevated position where he shone in the first rank, dying, it may almost be said, pen in hand, completing the scientific monument which will crown his long and laborious career. God who wills our destinies decided that the work to which the author had given the title of "*Forty years of Surgery*" should not be finished, or should be a posthumous production.

"M. ROUX leaves an imperishable name, a reputation which defies time and space. Friend and collaborator of BICHAT, he will participate in his honors and in the admiration accorded by posterity to his immortal genius.

"The obsequies of M. ROUX took place on Monday last at the Church of *Saint Germain des Prés.* The procession, composed of physicians, students, persons distinguished in science, letters and art, deputations from learned bodies, and teachers, increased at every step by the addition of groups of men and women who were waiting for its arrival to pay a debt of the heart to the memory of one who had attended them in sickness with a zeal and disinterestedness of which the poor will cherish a grateful remembrance. The vast nave of the church could scarcely contain the crowds who pressed towards it. The pall-bearers were MM. COMBES, President of the Academy of Sciences; PAUL DUBOIS, Dean of the Faculty of Medicine; DUBOIS, (d'Amiens,) perpetual Secretary of the Academy of Medicine; and DAVENNE, Director of Public Assistance.

"On leaving the Church a procession as large as before accompanied the mortal remains of the great surgeon to the cemetery of *Mont. Parnasse*, where they are to remain. There eloquent and impressive discourses were pronounced over the tomb by M. VELPEAU, in behalf of the Institute; by M. MALGAIGNE, in behalf of the Faculty of Medicine; by M. MARJOLIN, in behalf of the Society of Surgery; by M. FR. DUBOIS, in behalf of the Academy of Medicine; by M. LARREY, in behalf of the Military Surgeons; and by M. DUCHAUSSOY, in behalf of the Internes."

The funeral discourse of VELPEAU, which is given in full in the *Press Médicale*, is spoken of here as an eloquent and touching tribute to the memory of his distinguished *confrère*. It is thus spoken of the more because the author is not supposed to be much given to the melting mood. I quote the two closing paragraphs of his address, in which he uses the second person singular of the personal pronoun, a form of expression employed by the French in conversation with children only and very intimate friends:— "Venerated master, faithful friend, thou hast nothing to fear in a future life, thou hast well served thy fellow men, thou hast finished a long and noble career! Thy memory will never perish; thy name, ever honored, will occupy a large and beautiful page in the history of learned and useful men.

"From the abode of the elect, God will permit thy noble spirit sometimes to direct a kind look to us; thou wilt know that among your old pupils there is one at least who will cherish thy memory, and preserve his gratitude to thee even to his last moments; one who in bidding thee a last adieu, tears himself away from thy coffin with a heart swelling with emotions of sincere sorrow."

Such language from the lips of VELPEAU will be read with interest by those who have seen his impassive mien in his surgical wards and amphitheatre.

The summer course of instruction at the *Ecole de Médicine*, commenced with the first of April. Lectures are now given by CRUVEILHIER on Pathological Anatomy; GRISOLLE on Therapeutics, and REQUIN on Legal Medicine. Clinical courses are commencing at several of the hospitals. TROUSSEAU, at the *Hôtel Dieu*, began his course the day after my arrival. He is at the present moment the most popular medical clinical teacher in Paris. In his ward visits he is followed by a greater number than accompany any other hospital physician, and the amphitheatre, on the days of his lectures, (every other day,) is filled. At the Hospital *St. Antoine*, M. ARAN has commenced a clinical course, devoting on every Wednesday special attention to Auscultation and Percussion. M. PIORRY, at *La Charité*, is soon to commence a formal course. VELPEAU, BOUILLAUD, ANDRAL, RAYER, and BRIGUET are in daily attendance at *La Charité*. At the *Hôtel Dieu*, LUGOL and GRISOLLE are among those whose service is now going on. BARTH is on service at Hospital *Beaujon*, and at Hospital *St. Louis*, CAZENAVE and DEVERGIE. I give the names only of those whom I have seen. I shall write of certain hospitals and men more fully hereafter.

There is no lack of hospital opportunities. In fact they cannot all of them be improved simultaneously. The physician, or medical student, must select among the great number of hospitals and clinical teachers those of whom he desires to avail himself. The morning visits are made from 7 to 9 o'clock, A. M., and lectures, when they are given, follow directly the tour through the wards. This is before breakfast, the hour for the latter being in Paris from 10 to 12 o'clock. Visits in private practice are usually made after breakfast, or, in other words, in the afternoon, before dinner, which almost uniformly is at 9 o'clock, P. M. Admission to the hospitals during the hours of service is obtained without difficulty by any who are, or assume to be physicians. A person who bears a medical aspect passes without any question being asked, but if stopped by the *conceirge*, he is readily admitted on stating that he is a foreigner and a physician, or he is directed to the *bureau*, where on showing his passport, he receives a free ticket of admission, unlimited in time. With respect to freedom of access to all the hospitals for medical observation, nothing can be more liberal than the French system. If one wishes to visit and examine patients at other times than during the regular hour of service for the clinical professor, he must make an arrangement with an *interne*, who for a moderate fee, will at a different hour attend at the bedside, give any information that may be desired respecting the history, treatment, etc., of the cases, and at these visits any reasonable amount of examination of the cases is allowable. In this way special diseases may be studied to any extent. The *internes* are well educated physicians, perfectly conversant with the current and most recent facts and views in this city, and as the income from these services, though small, is generally a very convenient perquisite; they are anxious to have private classes and to make themselves as useful as possible. Many of the *internes* are abundantly competent to give clinical lectures, and they are much more minutely acquainted with the hospital cases than the clinical professors.

Of the habits of different clininal professors in hospital service I shall probably write hereafter. I will remark now that generally those at whose service I have been present make their examinations very hastily, devoting but a very brief space of time to the patients individually, relying mainly on the *internes.* In this particular I am surprised and disappointed. Except at the service of M. ARAN, at the Hospital *St. Antoine*, I have as yet seen no records of sypmtoms made at the bedside, nor any evidence that such records are made. The professors certainly do not themselves take notes of the cases, and if they are taken by the internes they are not produced at the time of the professors' service. The only record made at the bed-side pertains to the treatment, dietetic and medicinal. This fact takes away much of the value which, under other circumstances, we might attach to deductions from the large experience afforded by the hospitals of Paris.

In addition to the didactic course at the *Ecole de Médicine* and the clinical courses at the numerous hospitals, private and special courses on the different branches of medicine, surgery and obstetrics are constantly going on. At least a dozen courses of the latter description are at this moment advertised to commence within a short period. Among these is a course on microscopical anatomy, healthy and morbid, by M. ROBIN. M. BERNARD, who has lately been appointed professor, is soon to begin his course on experimental physiology. Of these two courses especially I shall probably, at a future time, give you some account. The health of Paris at the present time is excellent. The *Gazette des Hôpitaux* states that usually during the month of March and April acute inflammatory affections, such as pleurisy, pneumonia, and catarrh are prevalent; but this year they have been extremely unfrequent.

The hospital patients are for the most part affected with chronic maladies. No epidemic disease now exists, but epidemic cholera has but lately disappeared, and in this fact we have an explanation of the remarkable immunity from other epidemic, and also acute affections. From a bulletin contained in the journal just named, April 8, I find that from the 1st of March to the 5th of April there had been received into the hospital two hundred and six cases, the number of deaths being one hundred and eight.

In looking over the medical journals, issued in Paris during the time I have been here, I find nothing of special interest. I shall bear in mind to send you anything which may appear that I may suppose will interest yourself, and the readers of your journal.

The weather since my arrival has been delightful. There has been no rain, save a shower on one day. The foliage of the garden of the Tuilleries, opposite the open window at which I am seated, is luxuriant and rich, giving, at the early hour when I am writing, before the walks are crowded with people, the beauties of a rural prospect in the midst of this densely populated city.

With much esteem,

Yours truly,

AUSTIN FLINT.

